# Synthesis and electrochemical properties of copper(II), manganese(III) phthalocyanines bearing chalcone groups at peripheral or nonperipheral positions

**DOI:** 10.3906/kim-2006-21

**Published:** 2020-12-16

**Authors:** Hüseyin BAŞ, Nuran KAHRİMAN, Zekeriya BIYIKLIOĞLU*

**Affiliations:** 1 Department of Chemistry, Karadeniz Technical University, Trabzon Turkey

**Keywords:** Synthesis, peripheral, nonperipheral, copper, manganese, voltammetry

## Abstract

In this study, new chalcone compound **1** , new phthalonitrile derivatives **2** and **3,** new copper(II), manganese(III) phthalocyanines bearing chalcone groups at peripheral or nonperipheral positions were synthesized. Electrochemistry of tetra-(4-{(2 *E* )-3-[2-fluoro-4-(trifluoromethyl)phenyl]prop-2-enoyl}phenoxy) substituted Co(II)Pc and Mn(III)Pcs were studied with cyclic voltammetry (CV) to determine the redox properties of the phthalocyanines. According to the results, while the CuPcs **2a** and **3a** showed two Pc based reduction reactions and one Pc based oxidation reaction, MnPcs **2b** and **3b** gave two metal-based reduction reactions. All the redox processes are shifted toward positive potentials as a result of the increased electron-withdrawing ability of the trifluoromethyl substituents.

## 1. Introduction

Since pthalocyanines (Pcs) have chemical, thermal stability and an 18 p electron system, they have been very important derivatives for researchers [1]. In addition to their usage as paint, phthalocyanines are preferred as functional materials in different technologies, such as electrochemical sensors [2,3], solar cells [4,5], gas sensors [6,7], nonlinear optics [8], semiconductors [9,10], liquid crystals [11,12], photovoltaics [13], catalysts [14], electrochromics [15,16], and photosensitizers in photodynamic therapy (PDT) [17–19].

Electrochemical properties of phthalocyanines depend on several factors, such as the type of the central metal ions, substituents, and solvent [20,21]. Owing to these properties, phthalocyanines have been used in different electrochemical technologies. Chalcones are the common core scaffolds of many naturally occurring compounds, from ferns to higher plants [22]. Their skeleton structure and functional groups also make them well-known intermediates for the synthesis of various heterocyclic and bioactive compounds [23]. In literature it is shown that the introduction of chalcone groups into the peripheral/nonperipheral positions of phthalocyanines increases the electrochemical properties of phthalocyanines [24–28]. Also, phthalocyanines bearing chalcone groups have efficient photocatalytic, photophysical, and photochemical properties [29,30]. We wondered how the presence of phthalocyanines in the peripheral or nonperipheral positions of the chalcone group affects the electrochemical properties of phthalocyanine compounds. For this reason, in this work we combined these two functional compounds (chalcone and phthalocyanine) into a single compound. Finally, we synthesized copper(II), manganese(III) phthalocyanines bearing (4-{(2 *E* )-3-[2-fluoro-4-(trifluoromethyl)phenyl]prop-2-enoyl}phenoxy) groups at peripheral or nonperipheral positions and investigated their electrochemical properties.

## 2. Experimental

Materials, equipment, synthesis procedure, and electrochemistry experiments are given in the supplementary information.

## 3. Results and discussion

### 3.1. Synthesis and characterization

**Figure 1:**
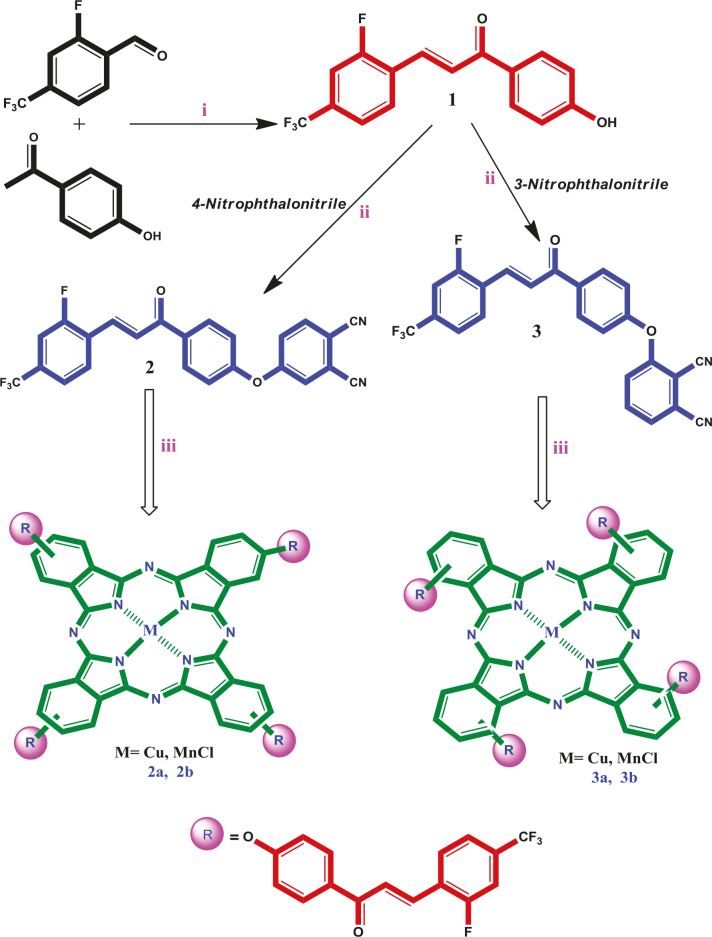
The synthesis of Cu(II)Pc and Mn(III)Pcs bearing (4-{(2E)-3-[2-fluoro-4-(trifluoromethyl)phenyl]prop-2-enoyl}phenoxy) groups. (i) NaOH, rt. (ii) DMF, K2CO3, 50 °C, 72 h. (iii) n-pentanol, DBU, 160 °C.

**Figure 2:**
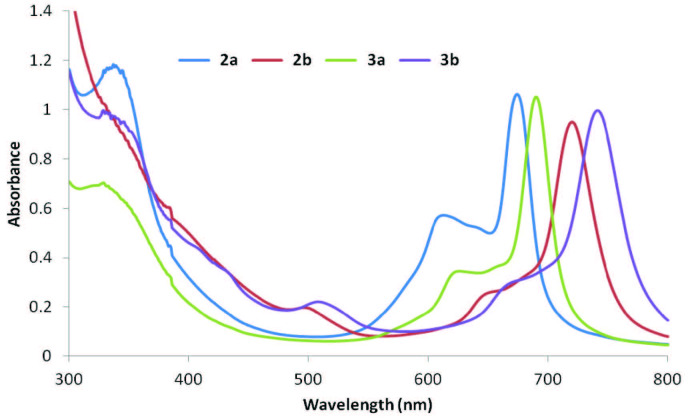
UV-Vis spectra of 2a, 2b, 3a, and 3b in THF. (Concentration: 1.00 × 10–5 M)

The synthesis of Pcs is given in Figure 1. Firstly, new chalcone compound (1) was obtained with the reaction between 2-fluoro-4-(trifluoromethyl)benzaldehyde and 4’-hydroxyacetophenone in alkaline conditions according to the known procedure of Claisen–Schmidt consendation [31]. Then, new phthalonitrile derivatives 2 and 3 were prepared with the reaction between chalcone compound (1) and 4-nitro- and 3-nitrophthalonitrile, respectively. Then, new peripheral and nonperipheral tetra substituted Cu(II) and Mn(III) Pc derivatives were synthesized with the reaction of corresponding nitril derivatives (2 and 3) and metal salt presence of DBU in 1-pentanol. Infrared spectra of compound 1 exhibited characteristic absorption bands of aliphatic C=C, C=O, and –OH groups at 1564 cm–1, 1653 cm–1, and 3328 cm–1, respectively. The most important evidence observed in the 1H-NMR spectrum of the formation of the chalcone compound is the peaks of the a α,β-unsaturated moiety. It was most clear with the vicinal coupling constant values (3JHα−Hβ = 16.0/16.0 Hz), that the configuration of these two protons was trans. Moreover, in the 13C-NMR, C-F coupling of aromatic and aliphatic carbons containing fluoro atoms doublets and multiplets peaks in the region of 163.1–108.2 ppm were formed. LC-MS/MS spectrum showed m/z = 311 [M+H]+ as a molecular ion peak. In the IR spectra of phthalonitrile compound (2), and phthalonitrile compound (3) the vibrations belonging to C=N groups were observed at 2234, and 2230 cm–1, respectively. In the 1H-NMR spectrum (2 and 3), the new aromatic protons demonstrated that phthalonitriles (2 and 3) were prepared. The m/z = 437 [M+H]+ mass peaks confirmed the structure of compounds (2 and 3). In the IR spectra of 2a, 2b, 3a, and 3b, sharp -C≡N vibrations of phthalonitrile compound (2), and phthalonitrile compound (3) disappeared as expected. Also, the IR spectra of the cobalt(II), manganese(III) Pcs (2a, 2b, 3a, and 3b) were very similar. The 1H-NMR, 13C-NMR spectra of 2a, 2b, 3a, and 3b could not be measured due to the paramagnetic nature [32]. In MALDI-TOF MS spectra of 2a, 2b, 3a, and 3b, the presence of molecular ion peaks at m/z = 1808.67 [M]+ for 2a, 1800.69 [M-Cl]+ for 2b, 1808.04 [M]+ for 3a, and 1800.78 [M-Cl]+ for 3b, confirmed the structures. On the UV-Vis spectra of peripheral or nonperipheral tetra-chalcone substituted cobalt(II), manganese(III) Pcs (2a, 2b, 3a, and 3b), the Q bands were observed as single and narrow bands at ~1 × 10–5 M (Figure 2). The absorption spectra of 2a, 2b, 3a, and 3b displayed Q bands at 674, 720, 690, and 741 nm. The B (Soret) bands of 2a, 2b, 3a, and 3b were observed at 337, 385, 329, and 330 nm. Furthermore, manganese(III) Pcs (2b, 3b) have an absorption band at 497 nm for 2b and 508 nm for 3b, interpreted as a charge transfer absorption [33].

### 3.2. Electrochemical studies

**Figure 3:**
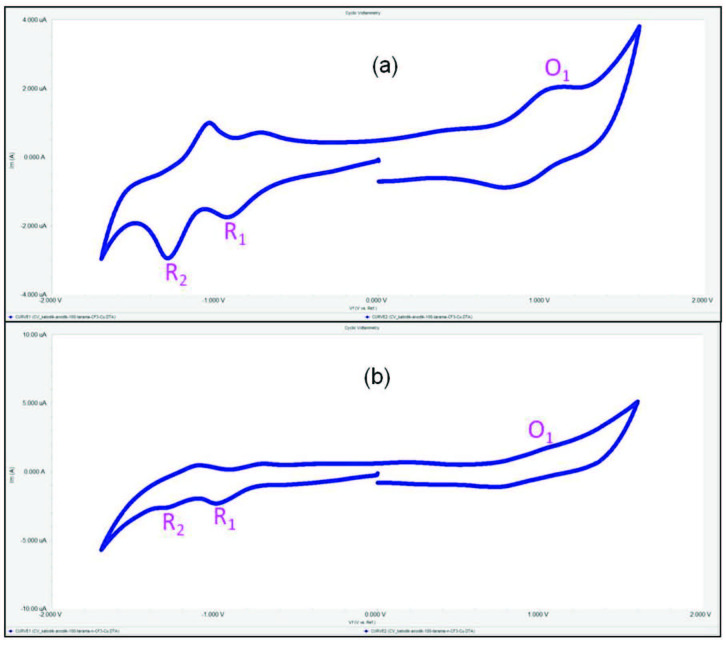
(a) CV graph of 2a, (b) CV graph of 3a.

**Figure 4:**
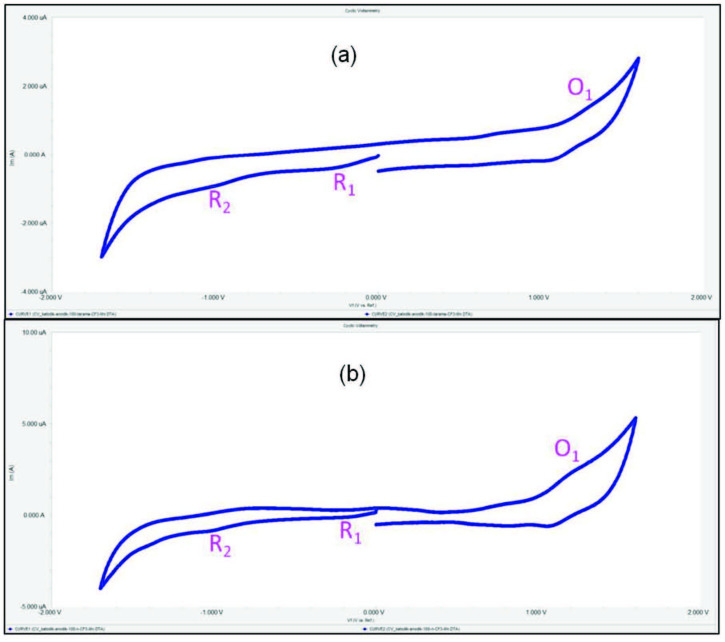
(a) CV graph of 2b, (b) CV graph of 3b.

Voltammetric responses of Pcs bearing (4-{(2 *E* )-3-[2-fluoro-4-(trifluoromethyl)phenyl]prop-2-enoyl}phenoxy) groups were carried out with CV in DCM/TBAP electrolyte on a Pt working electrode. The basic electrochemical data were tabulated in Table 1. Tetra-chalcone substituted copper(II) Pcs (2a, 3a) bearing trifluoromethyl groups have redox inactive metal centers. Increasing the electron-withdrawing ability of the substituents generally causes a positive shift of the redox processes [34]. For this reason, 2a and 3a showed only Pc oriented electron transfer reactions. But, tetra-chalcone substituted manganese(III) Pcs (2b, 3b) have redox active metal centers. For this reason, 2b and 3b showed metal based processes before the Pc processes. As shown in Figures 3a and 3b, tetra-chalcone substituted copper(II) Pcs (2a, 3a) showed two reversible (for 2a), two quasi-reversible (for 3a) Pc based reductions at –0.82 V (R1 for 2a), –0.85 V (R1 for 3a) for [CuIIPc2-]/[CuIIPc3-]1-, –1.16 V (R2 for 2a), –1.20 V (R2 for 3a) for [CuIIPc3-]1-/[CuIIPc4-]2- processes and one oxidation at 0.93 V (O1 for 2a), 0.88 V (O1 for 3a) processes. These electrochemical responses of tetra-chalcone substituted copper(II) Pcs (2a, 3a) are compatible with the literature [35]. Tetra-chalcone substituted manganese(III) Pcs (2b, 3b) showed two metal-based reduction reactions and one oxidation reaction. As shown in Figures 4a and 4b, R1 and R2 processes at –0.17 V (for 2b), –0.15 V (for 3b), –0.94 V (for 2b), and –0.96 V (for 3b) are assigned to [MnIIIPc2-]/[MnIIPc2-]1- and [MnIIPc2-]1-/[MnIPc2-]2- respectively. Also, during the anodic potential scans, a quasi-reversible Pc oxidation reaction appeared at 1.16 V (for 2b) and 1.13 V (for 3b), for [MnIIIPc2-]/[MnIIIPc1-]1+ couple. These results are in agreement with the literature [36]. Moreover, as shown in Table 1, all the redox processes are shifted toward positive potentials as a result of the increased electron-withdrawing ability of the trifluoromethyl substituents with respect to MnPc bearing (2-{2-[4-(1H-pyrrol-1-yl)phenoxy]ethoxy}ethoxy) groups [37], and CuPc bearing 2-(9,10-dioxo-9,10-dihydro-anthracen-2-yl-methyl)-malonic acid diethyl ester [38], synthesized in previous studies. When we compared the results, it is clear that binding the trifluoromethyl substituents to the peripheral or nonperipheral positions causes shifting of the peak potentials to the positive sides.

## 4. Conclusion

In this paper, copper(II), manganese(III) Pcs bearing chalcone groups at peripheral or nonperipheral positions were succesfully synthesized. The solubility of Pcs in organic solvents was increased by substitution of chalcone group to the peripheral or nonperipheral position of Pcs. Cyclic voltammetry was used to detect redox responses of Cu(II)Pcs and Mn(III)Pcs. Electrochemical measurements supported the suggested compounds. All Cu(II)Pcs and Mn(III)Pcs showed two reduction reactions during the cathodic potential scans. Results show that Mn(III)Pcs showed more redox activity than those of Cu(II)Pcs. The results demonstrated the usability of these Cu(II)Pcs and Mn(III)Pcs as functional materials owing to their rich redox responses. On the other hand, electrochemical measurements indicated that copper(II), manganese(III) Pcs bearing chalcone groups could be used for various electrochemical applications such as electrocatalytic, electrochromic and electrosensing.

**Table 1:** Voltammetric results of the Pcs. All voltammetric data were given versus SCE.

Pcs	Label	aE1/2	bDEp (mV)	cDE1/2
2a	R1	–0.82	98	1.75
	R2	–1.16	84	
	O1	0.93	88	
2b	R1	–0.17	134	1.33
	R2	–0.94	138	
	O1	1.16	140	
3a	R1	–0.85	123	1.73
	R2	–1.20	135	
	O1	0.88	140	
3b	R1	–0.15	138	1.28
	R2	–0.96	144	
	O1	1.13	140	

a: E1/2 values [(Epa+Epc)/2] were given versus SCE at 0.100 Vs–1 scan rate; b: ∆Ep= Epa-Epc; c: DE1/2 = E1/2 (first oxidation)–E1/2 (first reduction).

Supplementary Materials
