# Supplementary material for: Synthesis and electrochemical properties of copper(II), manganese(III) phthalocyanines bearing chalcone groups at peripheral or nonperipheral positions
Source: Turk J Chem. 2020 Dec 16;44(6):1549–55. doi: 10.3906/kim-2006-21 (PMC7765768; doi:10.3906/kim-2006-21)
Supplement: Supplementary file 1 — Supplementary Materials [file turkjchem-44-1549-sup001.pdf]

Supplementary information

1. Materials and equipment

4'-Hydroxyacetophenone and 2-fluoro-4-(trifluoromethyl)benzaldehyde were purchased from commercial suppliers. All reagents and solvents were of reagent grade quality and were obtained from commercial suppliers. The IR spectra were recorded on a Perkin Elmer 1600 FT-IR Spectrophotometer, using KBr pellets. ^{1}H and ^{13}C-NMR spectra were recorded on Bruker Avance III 400 MHz spectrometers in DMSO-d_6 and chemical shifts were reported (d) relative to Me_4Si as internal standard. Mass spectra were measured on a Micromass Quatro LC/ULTIMA LC-MS/MS spectrometer. MALDI-MS of complexes were obtained in dihydroxybenzoic acid as MALDI matrix using nitrogen laser accumulating 50 laser shots (Bruker Microflex LT MALDI-TOF mass spectrometer Bremen, Germany). Optical spectra in the UV-Vis region were recorded with a Perkin Elmer Lambda 25 spectrophotometer.

2. Synthesis

2.1. (2*E*)-3-[2-fluoro-4-(trifluoromethyl)phenyl]-1-(4-hydroxyphenyl)prop-2-en-1-one (1): To a stirred solution of 4'-hydroxyacetophenone (5 mmol) in ethanol (30 mL), 5 mL alcoholic NaOH (10 mmol) solution was added dropwise at room temperature. After mixing the reaction for 30 min, 2-fluoro-4-(trifluoromethyl)benzaldehyde (5 mmol) was added to the medium in portions and the reaction mixture was stirred for 16 h. Completion of reaction was monitored by TLC and after completion it was poured into water and acidified with HCl solution (1 N) until pH = 6–6.5 [1]. The obtained solid was filtered, washed with water, and dried in the lyophilizer. The purity of product was checked by TLC and supporting structural information was obtained using spectroscopic methods. Yield: 84%. M.p.: 146––148 °C. IR (ATR), ν/cm^{-1}: 3328 (O-H), 3081 (Ar-H), 1653 (C=O), 1564 (HC=CH), 1111 (Aromatic C-F), 817 (Aliphatic C-F). ^{1}H NMR (400 MHz, DMSO-d_6, ppm), (δ): 10.6 (bs, 1H, -OH), 8.4 (d, 1H, Ar-H), 8.1 (AB, *J*=16.0 Hz, 1H, =CH), 8.0 (d, 2H, Ar-H), 7.8 (d, 1H, Ar-H), 7.7 (AB, *J*=16.0 Hz, 1H, =CH), 7.6 (m, 1H, Ar-H), 6.9 (d, 2H, Ar-H). ^{13}C NMR (100 MHz, DMSO-d_6, ppm), (δ): 187.1, 163.1, 162.0, 159.5, 134.1, 132.6, 132.2, 132.0, 130.6, 130.5, 129.1, 129.0, 127.3, 127.1, 122.3, 122.1, 119.3, 116.0, 114.3, 114.0, 108.2. LC-MS/MS (ESI), (m/z): 311 $[M+H]^+$.

2.2. 4-(4-{(2*E*)-3-[2-fluoro-4-(trifluoromethyl)phenyl]prop-2-enoyl}phenoxy) phthalonitrile (2): (2*E*)-3-[2-fluoro-4-(trifluoromethyl)phenyl]-1-(4-hydroxyphenyl)prop-2-en-1-one **1** (0.8 g, 2.5 mmol) and 4-nitrophthalonitrile (0.45 g, 2.5 mmol) were dissolved in DMF (15 mL). Then, dry K_2CO_3 (1.1 g, 7.5 mmol) was added and stirred at 50 °C under N_2 atmosphere for 72 h. The mixture was cooled to room temperature and then poured into iced water (200 mL). After filtration under vacuum, the crude product was crystallized from ethanol. Yield: 0.44 g (40%), m.p. 159–160 °C. IR (ATR), ν/cm^{-1}: 3074 (Ar-H), 2921–2850 (Aliph. C-H), 2234 (C=N), 1665, 1603, 1587, 1564, 1502, 1484, 1428, 1329, 1275, 1247, 1210, 1165, 1111, 1067, 1009, 978, 854, 823, 743. ^{1}H NMR (400 MHz, DMSO-d_6, ppm), (δ): 8.40 (d, 1H, Ar-H), 8.31 (d, 2H, Ar-H), 8.18 (AB, 1H, =CH), 8.17 (d, 1H, Ar-H), 7.99 (d, 1H, Ar-H), 7.85 (AB, 1H, =CH), 7.84 (s, 1H, Ar-H), 7.73 (d, 1H, Ar-H), 7.60 (m, 1H, Ar-H), 7.37 (d, 2H, Ar-H). ^{13}C NMR (100 MHz, DMSO-d_6, ppm), (δ): 187.96, 162.16, 159.63, 159.05, 139.91, 136.96, 134.35, 132.20, 132.12, 130.78, 130.75, 130.74, 126.98, 126.92, 124.51, 124.09, 122.30, 122.20, 120.25, 120.09, 117.39, 116.31, 115.81, 114.40, 114.11, 110.02, 109.93. LC-MS/MS (ESI), (m/z): 437 $[M+H]^+$.

2.3. 3-(4-{(2*E*)-3-[2-fluoro-4-(trifluoromethyl)phenyl]prop-2-enoyl}phenoxy) phthalonitrile (3): Synthesized similarly to **2** from 3-nitrophthalonitrile. Yield: 0.39 g (35%), m.p. 184–185 °C. IR (ATR), ν/cm^{-1}: 3096 (Ar-H), 2921–2851 (Aliph. C-H), 2230 (C=N), 1666, 1611, 1596, 1572, 1504, 1456, 1430, 1330, 1275, 1217, 1172, 1162, 1110, 1069, 1033, 1007, 980, 911, 830, 803, 745. ^{1}H NMR (400 MHz, DMSO-d_6, ppm), (δ): 8.40 (t, 1H, Ar-H), 8.32 (d, 2H, Ar-H), 8.18 (AB, 1H, =CH), 7.97 (d, 1H, Ar-H), 7.92 (d, 1H, Ar-H), 7.84 (AB, 1H, =CH), 7.83 (d, 1H, Ar-H), 7.72 (d, 1H, Ar-H), 7.54 (m, 1H, Ar-H), 7.41 (d, 2H, Ar-H). ^{13}C NMR (100 MHz, DMSO-d_6, ppm), (δ): 187.91, 162.14, 159.62, 159.23, 158,77, 136.71, 134.41, 133.93, 133.89, 132.09, 130.76, 130.72, 130.31, 129.92, 126.96, 126.91, 124.38, 123.33, 122.35, 122.27, 119.81, 116.63, 16.04, 114.40, 114.10, 113.67, 113.60, 107.06. LC-MS/MS (ESI), (m/z): 437 $[M+H]^+$.

2.4. 2(3),9(10),16(17),23(24)-Tetrakis-(4-{(2*E*)-3-[2-fluoro-4-(trifluoromethyl)phenyl]prop-2-enoyl}phenoxy)-phthalocyaninato copper(II) (2a): 4-(4-{(2*E*)-3-[2-fluoro-4-(trifluoromethyl)phenyl]prop-2-enoyl}phenoxy) phthalonitrile (2) (100 mg, 0.23 mmol), $CuCl_2$ (16 mg, 0.11 mmol), 1-pentanol (2 mL), 1,8-diazabicyclo[5.4.0]undec-7-ene (DBU) (3 drops) was stirred at 160 °C for 24 h. The mixture was precipitated with ethanol. The green product was obtained by column chromatography using basic aluminum oxide and $CHCl_3$ as eluent. Yield: 25 mg (25%), m.p. > 300 °C. IR (ATR), ν/cm^{-1}: 3066 (Ar-H), 2918–2849 (Aliph. C-H), 1682, 1595, 1504, 1463, 1429, 1328, 1259, 1235, 1164, 1121, 1092, 1012, 950, 876, 800. UV-Vis (THF) λ_{max} nm (log e): 674 (4.02), 613 (4.75), 337 (5.07). MALDI-TOF-MS m/z: 1808.67 $[M]^+$.

2.5. 2(3),9(10),16(17),23(24)-Tetrakis--(4-{(2*E*)-3-[2-fluoro-4-(trifluoromethyl)phenyl]prop-2-enoyl}phenoxy)-phthalocyaninato manganese(III)chloride (2b) : Synthesized similarly to 2a by using $MnCl_2$ instead of $CuCl_2$. Yield: 21

mg (20%), m.p. > 300 °C. IR (ATR), ν/cm^{-1} : 3078 (Ar-H), 2918–2849 (Aliph. C-H), 1684, 1595, 1526, 1504, 1463, 1407, 1330, 1259, 1234, 1165, 1119, 1072, 1013, 954, 910, 797. UV-Vis (THF) λ_{max} nm (log e): 720 (4.97), 652 (4.41), 497 (4.29), 385 (4.78). MALDI-TOF-MS m/z: 1800.69 [M-Cl]$^+$.

2.6. 1(4),8(11),15(18),22(25)-Tetrakis--(4-{(2*E*)-3-[2-fluoro-4-(trifluoromethyl)phenyl]prop-2-enoyl}phenoxy)-phthalocyaninato copper(II) (3a) : Synthesized similarly to 2a by using 3-(4-{(2*E*)-3-[2-fluoro-4-(trifluoromethyl) phenyl]prop-2-enoyl}phenoxy)phthalonitrile (3) instead of 4-(4-{(2*E*)-3-[2-fluoro-4-(trifluoromethyl)phenyl]prop-2-enoyl}phenoxy)phthalonitrile (2). Yield: 22 mg (22%), m.p. > 300 °C. IR (ATR), ν/cm^{-1} : 3068 (Ar-H), 2919–2850 (Aliph. C-H), 1680, 1582, 1503, 1483, 1427, 1328, 1244, 1214, 1164, 1120, 1089, 1065, 1006, 911, 877, 799, 743. UV-Vis (THF) λ_{max} nm (log e): 690 (5.02), 625 (4.53), 329 (4.4). MALDI-TOF-MS m/z: 1808.04 [M]$^+$.

2.7. 1(4),8(11),15(18),22(25)-Tetrakis--(4-{(2*E*)-3-[2-fluoro-4-(trifluoromethyl)phenyl]prop-2-enoyl}phenoxy)-phthalocyaninato manganese(III)chloride (3b) : Synthesized similarly to 2b by using 3-(4-{(2*E*)-3-[2-fluoro-4-(trifluoromethyl)phenyl]prop-2-enoyl}phenoxy)phthalonitrile (3) instead of 4-(4-{(2*E*)-3-[2-fluoro-4-(trifluoromethyl) phenyl]prop-2-enoyl}phenoxy)phthalonitrile (2). Yield: 18 mg (17%), m.p. > 300 °C. IR (ATR), ν/cm^{-1} : 3073 (Ar-H), 2927–2857 (Aliph. C-H), 1682, 1580, 1503, 1484, 1424, 1327, 1242, 1211, 1163, 1138, 1067, 1010, 984, 911, 825, 743. UV-Vis (THF) λ_{max} nm (log e): 741 (4.99), 670 (4.47), 508 (4.34), 330 (4.99). MALDI-TOF-MS m/z: 1800.78 [M-Cl]$^+$.

3. Electrochemical measurements

The cyclic voltammetry (CV) and square wave voltammetry (SWV) measurements were carried out with Gamry Interface 1000 potentiostat/galvanostat controlled by an external Pc and utilizing a three-electrode configuration at 25 °C. The working electrode was a Pt disc with a surface area of 0.071 cm^2. A Pt wire served as the counter electrode. Saturated calomel electrode (SCE) was employed as the reference electrode and separated from the bulk of the solution by a double bridge. Electrochemical grade TBAP in extra pure DCM was employed as the supporting electrolyte at a concentration of 0.10 mol dm^{-3}.

References

1. Yaylı N, Küçük M, **Üçüncü** O, Yaşar A, Yaylı et al. Synthesis of N-alkyl derivatives and photochemistry of nitro (E)-3-azachalcones with theoretical calculations and biological activities. Journal of Photochemistry and Photobiology A-Chemistry 2007; 188: 161-168.
